# What is in a name? Rethinking SMUG1 in genome maintenance

**DOI:** 10.1093/narcan/zcaf050

**Published:** 2025-12-12

**Authors:** Natalie Rudolfova, Alexander Myhr Skjetne, Nicola P Montaldo, Torkild Visnes, Hilde Loge Nilsen, Maurice Michel

**Affiliations:** Department of Oncology and Pathology, Science for Life Laboratory, Karolinska Institute, Stockholm, Solna, 17165, Sweden; Center for Molecular Medicine, Karolinska Institute and Karolinska Hospital, Stockholm, 17176, Sweden; Department of Microbiology, Oslo University Hospital, Norway; Institute of Clinical Medicine, University of Oslo, Norway; CRESCO—Centre for Embryology and Healthy Development, University of Oslo, 0424 Oslo, Norway; Department of Microbiology, Oslo University Hospital, Norway; Institute of Clinical Medicine, University of Oslo, Norway; CRESCO—Centre for Embryology and Healthy Development, University of Oslo, 0424 Oslo, Norway; Department of Biotechnology and Nanomedicine, SINTEF Industry,7034 Trondheim, Norway; Department of Microbiology, Oslo University Hospital, Norway; Institute of Clinical Medicine, University of Oslo, Norway; CRESCO—Centre for Embryology and Healthy Development, University of Oslo, 0424 Oslo, Norway; Department of Oncology and Pathology, Science for Life Laboratory, Karolinska Institute, Stockholm, Solna, 17165, Sweden; Center for Molecular Medicine, Karolinska Institute and Karolinska Hospital, Stockholm, 17176, Sweden

## Abstract

Small base lesions in DNA are primarily repaired through the base excision repair pathway, which is initiated by DNA glycosylases. This review focuses on single-strand selective monofunctional uracil–DNA glycosylase (SMUG1), an enzyme whose name incompletely captures its broader biological roles. SMUG1 excises a wide range of substrates beyond uracil, shows a preference for double-stranded DNA, and has been reported to be a bifunctional DNA glycosylase with a weak lyase activity. Moreover, SMUG1 plays roles extending beyond DNA repair, including functions in RNA quality control and RNA biogenesis. Recently, genetic interactions have been described between SMUG1 and proteins that safeguard stressed replication forks, implicating a function for SMUG1 in cancer cell biology. Understanding SMUG1’s full repertoire is key to uncovering its role in genome maintenance and unlocking its potential as a therapeutic target. Here, we review the biochemical properties reported for SMUG1 and its distinct functions from other uracil–DNA glycosylases *in vivo*. We also highlight the emerging role of SMUG1 in cancer cells and its potential as a therapeutic target, emphasizing the need to define the genetic and molecular contexts in which its modulation may be beneficial.

## Graphical Abstract

**Graphical Abstract:**
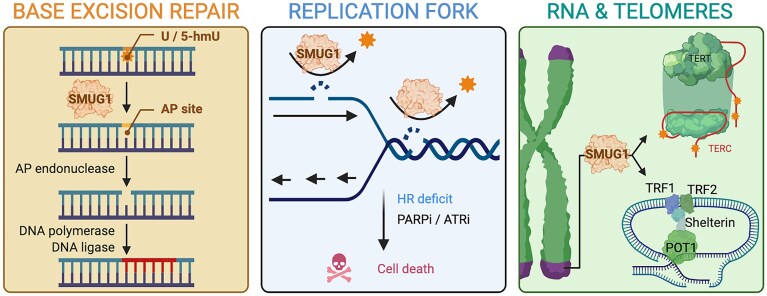

## Introduction: endogenous DNA damage and base excision repair

DNA is the carrier of genetic information and thus is one of the most important biological macromolecules. DNA is vulnerable to damage, and cells rely on intricate repair mechanisms to preserve its integrity. When these mechanisms fail, it can lead to mutagenesis and carcinogenesis. In 2015, Tomas Lindahl, Paul Modrich, and Aziz Sancar were awarded the Nobel Prize in Chemistry for their mechanistic studies of DNA repair. Lindahl and others showed that the instability of DNA is not only due to exogenous factors but also due to spontaneous decomposition events such as depurination, deamination, and oxidation. Depurination leads to the formation of an apyrimidinic/apurinic site (AP site), which is prone to single-strand break *via* the β-elimination reaction, creating progressively more toxic structures.

Uracil and other lesions such as 5-hydroxymethyluracil (5-hmU) and 5-formyluracil (5-fU) may form in DNA spontaneously but also through enzymatic reactions that evolved to alter DNA in a controlled manner. Cytosine, for example, can be deaminated by activation-induced cytidine deaminase/apolipoprotein B mRNA-editing enzyme catalytic polypeptide-like (AID/APOBEC) family enzymes, leading to the formation of uracil [1], potentially leading to a C-to-T transition—a common spontaneous mutation found in human tumour cells [2]. This family of enzymes defines an innate immune response involving viral restriction and RNA editing [2]. In mammals, several cytidine deaminases work on DNA, with AID being important in modulation of immunoglobulin genes in somatic hypermutation and class switch recombination—processes needed for generation of high-affinity antibodies [3], whereas the APOBEC catalytic subunit 3A (A3A) and APOBEC catalytic subunit 3B (A3B) enzymes function in tumour restriction and evolution [2]. In addition, the Ten-Eleven Translocation (TET) family of enzymes modifies DNA bases, primarily converting 5-methylcytosine (5-mC) to 5-hydroxymethylcytosine (5-hmC) and further to 5-carboxylcytosine (5-caC) and 5-formylcytosine (5-fC) through oxidation. TET enzymes can also oxidize T forming 5-hmU [4].

**Figure 1.**
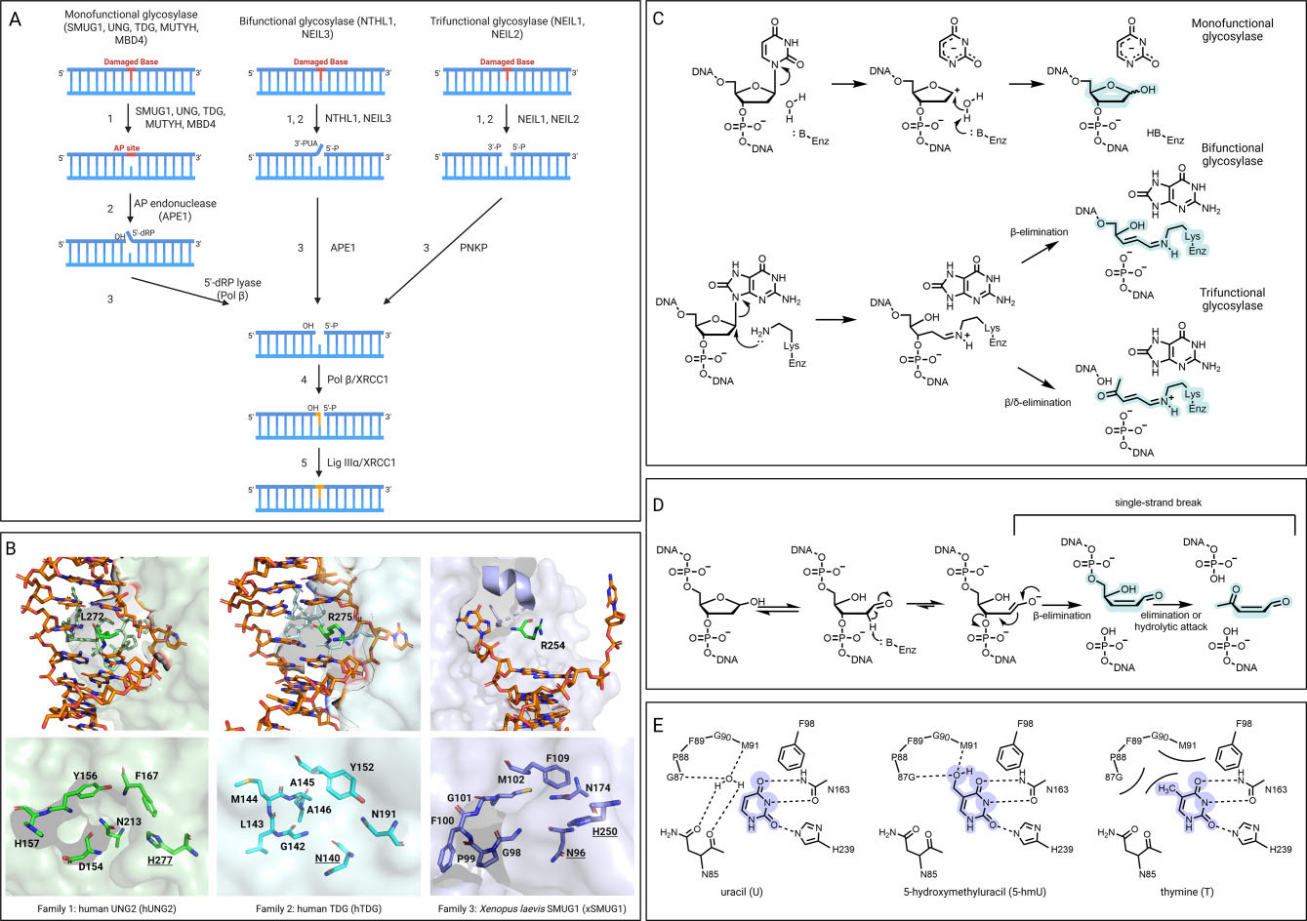
Molecular mechanisms of DNA glycosylases, structure, and chimeric active site of SMUG1. (**A**) Overview of the BER pathway. The repair process is initiated by a DNA glycosylase and followed by several other enzymes depending on the type of the glycosylase. The process can be divided into five steps: (i) base excision, (ii) DNA strand incision, (iii) processing of the nicked strands, (iv) synthesis of new nucleotides, and (v) ligation of the DNA strand. (**B**) Even though SMUG1 binds to the end of DNA oligonucleotide in the crystal structure (PDB: 1OE5) [6], it is apparent that the DNA duplex is more distorted than in the structure of DNA with uracil–DNA glycosylase (UNG) (PDB: 1SSP) [7] or TDG (PDB: 5HF7) [8]. DNA is in orange in all figures. In hUNG2 and hTDG, L272 and R275, respectively, are inserted into the duplex (in green). In the crystal structure of xSMUG1, a wedge is inserted into the duplex, and both bases are flipped out. The active site of xSMUG1 is a chimera of active sites of hUNG2 and hTDG. The residue His250 in SMUG1 responsible for activation of water molecule is conserved in hUNG2 (H268), and the Asn96 is conserved in hTDG (N140). (**C**) Molecular mechanisms of reactions catalysed by mono-, bi-, and trifunctional glycosylases. Bi- and trifunctional glycosylases have an additional AP-lyase activity catalysing a β- and β/δ-elimination, respectively. (**D**) SMUG1 was reported to catalyse strand incision, but since borohydride trapping experiments did not produce cross-linked enzyme–DNA product, a mechanism involving an enolate intermediate was proposed. (**E**) hSMUG1 is assumed to discriminate between uracil and thymine thanks to a well-ordered water molecule [9].

Damaged or non-canonical DNA bases are primarily repaired by the base excision repair (BER) pathway. Generally, this process is initiated by a DNA glycosylase and can be divided into several steps: excision of a damaged or misincorporated base, incision of the DNA backbone or processing of the resulting AP site, incorporation of the missing nucleotide by DNA polymerase, and lastly, ligation of the DNA strand by a ligase (Fig. 1A) [5].

DNA glycosylases can be divided into three categories according to function. Monofunctional glycosylases, such as uracil–DNA glycosylase (UNG), SMUG1, thymine–DNA glycosylase (TDG), and methyl–CpG binding domain 4 (MBD4), recognize and excise the damaged base, leaving an AP site. Bifunctional (endonuclease VIII-like 3, NEIL3; endonuclease III-like protein 1, NTHL1) and trifunctional (endonuclease VIII-like 1&2, NEIL1&2) DNA glycosylases also possess AP-lyase activity, with the former catalysing β-elimination and the latter both β- and δ-eliminations upon base excision [10]. There are at least four DNA glycosylases that can excise uracil in humans: UNG (of which there are two isoforms—UNG1 and UNG2, mainly localized in mitochondria and the nucleus, respectively [11–14]); TDG [15]; SMUG1 [16]; and MBD4 [17]. Although uracil is a substrate for all of these enzymes *in vitro*, their biochemical properties, substrate spectra, regulation, and intracellular localization differ. It can therefore be assumed that their specific roles in uracil repair are somewhat different [18]. The context in which uracil arises in DNA, its location in the genome, cell-cycle stage, or tissue, might determine which DNA glycosylase initiates the repair process [19–21].

This review focuses on SMUG1, which was initially suggested to be a broad-specificity backup for UNG [20]. SMUG1’s preference for double-stranded DNA substrates challenges its designation as being single-stranded selective. The roles of SMUG1 beyond classical BER [22–29] (summarized by Lirussi *et al. *[30]) highlight its functional distinction from other UNGs. Here, we provide an overview of the current knowledge of SMUG1 in DNA repair—from its remarkable active site that can accommodate a wide range of lesions, its role in BER, emerging evidence of functions in cancer cells.

## SMUG1 biochemical properties

SMUG1 was first identified over 40 years ago by Hollstein *et al*. as a 5-hydroxymethyluracil-DNA glycosylase (HMUDG) in mouse plasmacytoma cells [31]. It was later shown to be distinct from UNG and independently cloned from Xenopus by Haushalter *et al*., who named it SMUG1 based on its substrate specificity [16, 32]. Subsequent studies confirmed that HMUDG, SMUG1, and other related glycosylase activities (e.g. FDG) are all attributed to the same enzyme (reviewed by Raja and Van Houten [33]).

The UNG structural superfamily consists of several families. Even though the sequence identity between different families is small, they share a characteristic α/β fold structural motif [34]. This motif was readily apparent in the first published crystal structure of SMUG1 from *Xenopus laevis*, xSMUG1, which thus became the founding member of family 3 of the UNG superfamily [6].

In the co-crystal structure of xSMUG1 and a DNA oligonucleotide, SMUG1 was found binding cytosine at the end of the double-stranded DNA (dsDNA) after uracil excision. Although the cytosine was flipped out of the double helix, it did not bind to the active site, and thus SMUG1 was captured in a non-active conformation [6]. Like the majority of DNA glycosylases, SMUG1 flips out the aberrant base through the major groove; however, unlike UNG [7] or TDG [8], it inserts a wedge into the duplex, causing greater distortion of the DNA helix while forming extensive interactions with the opposite strand (Fig. 1B) [6]. SMUG1 was shown to prefer excision of uracil and 5-hmU when paired to guanine as opposed to adenine—this could be explained by these extensive interactions with the opposite strand [6, 35, 36, 9].

When the xSMUG1–DNA crystals were soaked with uracil, a structure of xSMUG1 with uracil bound in the active site was obtained [6]. The active site was found to be a chimera of family 1 and 2 UNGs—having the active histidine residue of UNG and the asparagine of the prokaryotic counterpart of TDG, mismatch-specific uracil–DNA glycosylase (MUG) (Fig. 1B, reviewed by Raja and Van Houten [33]). While the histidine residue is assumed to stabilize a transition state during catalysis, the asparagine seemingly coordinates the water molecule in a favourable position for the nucleophilic attack [9]. Distinction between thymine and uracil is facilitated by a well-ordered water molecule present in the active site (Fig. 1E) [6]. A similar interaction with a water molecule was observed in the co-crystal of the *Geobacter metallireducens* SMUG1 (GmeSMUG1) with xanthine [37]. This water molecule is only needed for the binding of uracil and not substrates with a hydrophilic substituent at C5 (Fig. 1E). This different mode of binding is consistent with the G90A SMUG1 mutant displaying complete loss of activity towards uracil but not for 5-hydroxyuracil (5-hU), 5-hmU, and 5-fU [9]. The side chain of A90 in this mutant displaces the water molecule, thus preventing uracil from binding. To our knowledge, a crystal structure of human SMUG1 (hSMUG1) or the hSMUG1–DNA complex in a productive conformation has not been solved.

Interestingly, the active site of SMUG1 can accommodate a wide range of base lesions. These lesions include both purines and pyrimidines that can arise in DNA *via* various processes summarized in Table 1. However, there have been conflicting results regarding some of the SMUG1 substrates in the literature.

**Table 1.** Reported substrates of SMUG1 and their origins in DNA

Base lesion	Origin in the DNA
Uracil (U) [16]	Misincorporation of deoxyuridine triphosphate, hydrolytic, or enzymatic deamination of cytosine
5-hydroxymethyluracil (5-hmU) [35]	Reactions of thymine with hydroxyl radicals, enzymatic oxidation, or misincorporation of 5-hydroxymethyluracil triphosphate
5-carboxyuracil (5-caU) [38]	Reactions of thymine with hydroxyl radicals and enzymatic oxidation
5-formyluracil (5-fU) [39]	Reactions of thymine with hydroxyl radicals and enzymatic oxidation of thymine
5-hydroxyuracil (5-hU) [39]	Reactions of cytosine with hydroxyl radicals, enzymatic oxidation, and deamination of cytosine
Alloxan [40]	Reactions of cytosine with hydroxyl radicals
Isodialuric acid [40]	Reactions of cytosine with hydroxyl radicals
3, *N*^4^-ethenocytosine (εC) [20, 41]	Reaction of metabolism products of vinyl chloride with cytosine or endogenously by reactions of lipid peroxidation products with cytosine
5-fluorouracil (5-FU) [20, 42, 43]	Does not appear in DNA naturally
5-bromouracil (5-BrU) [44]	Enzymatic bromination of uracil
5-iodouracil (5-IU) [44]	Does not appear in DNA naturally
Oxanine [45]	Hydrolytic and enzymatic deamination of guanine
Xanthine [46]	Hydrolytic and enzymatic deamination of guanine

SMUG1 can excise a wide range of lesions, including both purine and pyrimidine bases. The base lesions can arise in the DNA via spontaneous or enzymatic processes.

Kavli *et al*. [20] reported that SMUG1 cannot excise 5-hU, which is in contrast to several other published studies [38–40, 47]. The experiments by Kavli *et al*. were done in the presence of AP-endonuclease 1 (APE1), the enzyme catalysing DNA incision after base excision by SMUG1 (see Fig. 1A), and Mg^2+^, and the identity of the SMUG1 enzyme assayed was verified by inhibiting antibodies [20]. The hydroxy group in 5-hU is ionizable with a pK_a_ value close to the physiological pH [38]; thus, a slight deviation in pH during the experiment might change the ability of SMUG1 to excise this substrate and hence lead to conflicting results.

Similarly, Wibley *et al*. [6] reported that SMUG1 did not excise 3, *N*^4^-ethenocytosine (εC), which was reported as a substrate by Kavli *et al*. [20] and later by Goto *et al*. [41], who showed that SMUG1 was able to excise εC paired to adenine, thymine, cytosine, and guanine. The cleavage assay utilized by Wibley *et al*. was run without APE1 (unlike Kavli *et al*.); however, this is unlikely to be the reason for the different observations, as the assay run by Goto *et al*. also did not include this enzyme. Wibley *et al*. used a truncated version of xSMUG1, lacking the first 23 amino acids at the N-terminus corresponding to the first 13 residues in the human sequence. It is unlikely that the truncation changed the reactivity since the enzyme was still active towards uracil and 5-hmU [6].

Although Haushalter *et al. *[16] first reported SMUG1 selective for single-stranded DNA (ssDNA), several other studies have since shown that this selectivity is influenced by the reaction conditions [15, 16, 47, 48]. SMUG1, like other DNA glycosylases, seems to be end-product inhibited [19, 48]. Kinetic studies suggest that the excision-product dissociation is the rate-limiting step [49, 50]. SMUG1 binds to AP sites more tightly than to uracil in dsDNA [48–50]; however, this was not observed for uracil in ssDNA, and it is unclear whether this relationship also stands for the other substrates [20, 50].

*In vivo*, there are many proteins involved in BER, which might modulate each other’s activities [51, 52]. For example, the APE1 endonuclease can enhance the activity of various DNA glycosylases [53, 54]. Multiple studies reported that the presence of both APE1 and Mg^2+^ increases SMUG1 selectivity for dsDNA, along with increasing its activity [19, 20, 55, 50]. Mg^2+^ is needed for the catalytic activity of APE1 but not for DNA binding [56], and APE1 was shown to have similar turnover rates on ds- and ssDNAs [57]. Doseth *et al*. reported that the addition of APE1 alone did not modulate the reactivity or activity of uracil in ssDNA or dsDNA, and it seems that catalytically active APE1 is needed for stimulation of activity [55].

Conflicting data exist with respect to the effect of metal ions on SMUG1 activity. Since Mg^2+^ has been shown to modulate activity and substrate specificity of other DNA glycosylases, its effects on SMUG1 have been investigated. Kavli *et al*. showed that the addition of Mg^2+^ (7.5 mM) increases the activity towards uracil in both ssDNA and dsDNA [20], while Doseth *et al*. reported that the addition of Mg^2+^ (7.5 mM) increased the activity towards uracil in dsDNA (paired to guanine) while the activity decreased towards uracil in ssDNA [55]. Both experiments were done with excess substrate and with NaCl (10 mM) in the reaction mixture, but the former was done at 30°C for 10 min, while the latter at 37°C for 20 min.

Masaoka *et al*. [39] demonstrated that at low NaCl concentrations (3 mM), SMUG1 is single-strand selective for uracil, 5-hU, 5-hmU, and 5-fU but double-strand selective at high concentrations of NaCl (50 mM). There was no Mg^2+^ or APE1 in these reaction mixtures. These findings contradict the first SMUG1 paper by Haushalter *et al*., where it was reported that at 50 mM NaCl SMUG1 was ssDNA selective [16]. At higher salt concentrations, the DNA double helix is more compact and rigid [58], and it is possible that this influences the ability of SMUG1 to insert its wedge into the DNA duplex, flip out the damaged base, and excise it. Overwhelmingly, SMUG1 has been assayed at hypotonic salt concentrations, suggesting that the activity observed at higher salt concentrations may be more biologically relevant.

More studies are needed to fully elucidate the effects of reaction conditions on SMUG1 base excision activity. It will be interesting to follow whether advancements in single-molecule analyses can shed light on SMUG1’s enzymatic properties, such as the effects of monovalent and bivalent ions, AP site affinity, and whether SMUG1 handles all substrates in the same way.

## Monofunctional, bifunctional, or trifunctional DNA glycosylase

There are three classes of DNA glycosylases, distinguished by the reactions they catalyse (Fig. 1C). Monofunctional glycosylases such as SMUG1 excise the substrate base and leave an AP site. AP sites are unstable, especially under alkaline conditions, and their decomposition can lead to SSBs [5]. SSBs are cytotoxic lesions that are detrimental to the cell [59]. SMUG1 binds tightly to the AP site, competing with APE1, and this might be a mechanism protecting cells by preventing conversion of the repair intermediate to SSB [50].

Cannon-Carlson *et al*. reported that the purest fractions of HMUDG (SMUG1) isolated from calf thymus had both DNA glycosylase and Mg^2+^-dependent AP-endonuclease activities, which could be attributed to the same protein due to similar thermal activations [32]. These reactions were run for 5 h, and it is possible that even a very small contamination, such as a nuclease, would be able to incise the DNA backbone during that time. Matsubara *et al*. reported faint bands in their gels attributed to the nicked product when characterizing FDG (SMUG1) [47]. Despite these observations, the authors concluded that the nicked product was not due to enzymatic activity but rather spontaneous decomposition.

Alexeeva *et al*. reported that SMUG1 incises the DNA strand subsequent to uracil excision [60]. They observed a DNA incision product after 10 min of reaction at 37°C and without Mg^2+^ to ensure zero APE1 activity. Nevertheless, they reported that SMUG1 was able to catalyse the β- and δ-elimination reactions, suggesting that SMUG1 may act as a trifunctional glycosylase. However, they observed the incision product without adding Mg^2+^ to the reaction [60], which directly contrasts with Cannon-Carlson *et al*. [32].

Monofunctional glycosylases catalyse the excision of a substrate base via an activated water molecule, while bifunctional and trifunctional glycosylases utilize an amine group (Fig. 1C). The latter reaction proceeds via a Schiff base intermediate, which can be trapped by reaction with NaBH_4_. However, Alexeeva *et al*. found no cross-linked DNA-enzyme product upon treatment with NaBH_4_, suggesting that this reaction proceeds via a different mechanism [60]. Thus, the authors proposed that the excision and incision reactions were not concerted and that the latter proceeds via an enolate intermediate (Fig. 1D) [60]. It is not clear what residue is the general base, possibly His239, shown to be necessary for the base excision reaction, nor what residues stabilize the enolate intermediate. Independent confirmation is clearly needed to conclude whether SMUG1 acts as a monofunctional, bifunctional, or even trifunctional glycosylase.

It is worthwhile to point out that the classification of DNA glycosylases into any of the functionalities has led to quite some confusion in the literature. Like OGG1, which is a monofunctional DNA glycosylase in cells but has been observed to act as a bifunctional DNA glycosylase *in vitro *[61, 62], neither SMUG1 nor UNG2 have been observed to incise the DNA backbone *in cellulo*. It is therefore more suitable to speak of an enzymatic activity when measured in biochemical assays *in vitro* and of enzymatic function when assessed in a living cell or *in vivo*.

## SMUG1 and UNG: shared and distinct functions *in vivo*

There are at least four enzymes able to excise uracil in humans, each likely evolved to offer unique functions and roles, further stressing the importance of uracil repair in DNA [63]. TDG [64] and MBD4 [17] are able to remove deaminated cytosines in methylated CpG sites, and TDG seems to be the only UNG necessary for embryogenesis [65]. TDG-mediated base excision repair initiates cell fate specification programs in neuronal and macrophage development by creating DNA single-strand breaks at TET-induced base modifications within enhancers [66].

SMUG1 is the main DNA glycosylase responsible for excision of 5-hmU [67]. While 5-hmU is not a substrate for UNG, uracil is a substrate for both SMUG1 and UNG [20]. SMUG1, unlike UNG, does not interact with replication protein A (RPA) and does not have a proliferating cell nuclear antigen binding domain. SMUG1 is also more efficient at lower concentrations of substrate than UNG [19]. SMUG1 is expressed at relatively uniform levels throughout the cell cycle [20], whereas UNG protein is cell-cycle regulated with its highest levels in the S-phase [20, 68], and the UNG2 protein is actively degraded in G2 phase [69]. Similarly, tissues with high cell turnover tend to have higher UNG expression, whereas SMUG1 expression and activity are higher in tissues with lower turnover, such as the brain [21, 67]. Collectively, these observations support that these DNA glycosylases might have different primary functions and that the assessment of SMUG1 function *in vivo* should be studied without wearing UNG goggles.

The hypothesis that SMUG1 might specialize in excision of deaminated cytosines rather than of *de novo* incorporated uracils during replication [19] is not supported by *in vivo* evidence. Instead, the synergistic increase in uracil levels when both UNG2 and SMUG1 are perturbed suggests some functional overlap with respect to uracil BER *in vivo *[21]. An anti-mutator role of SMUG1 was suggested, as siRNA-mediated knockdown of SMUG1 in UNG-knockout mouse embryogenic fibroblasts (MEFs) gave an additive increase in the spontaneous mutant frequency using the hypoxanthine phosphoribosyl transferase assay [40]. Later, mutation accumulation lines supported that UNG contributed more than SMUG1 to counteract genome-wide spontaneous mutations in MEFs [70]. A combined deficiency of both SMUG1 and UNG, however, showed a synergistic effect, with an ~10-fold increase in spontaneous C-to-T transitions at non-CpG sites [40]. These findings highlight the distinct and nonredundant roles of SMUG1 and UNG in limiting cytosine deamination-induced mutagenesis.

In contrast to mice, UNG-deficient bacteria and yeast have much greater rates of mutations than their wild-type counterparts [71, 72]. These observations may be due to the lack of SMUG1 since vertebrates have both UNG and SMUG1 enzymes, while yeast and bacteria have only one of the two [50]. Elateri *et al*. reported that SMUG1 can compensate for UNG in yeast *Saccharomyces cerevisiae *[73], whereas Pettersen *et al*. reported that this is not the case in *Escherichia coli *[50]. These contrasting results might be due to different experiment conditions used: Elateri *et al*. used an antifolate treatment, which leads to S-phase arrest, whereas Pettersen *et al*. introduced U:G lesions by overexpressing AID [73]. In the latter study, expressing catalytically active AID and SMUG1 impaired growth, suggesting that SMUG1 can excise uracil and generate cytotoxic AP sites [73] but somehow inhibits productive repair [50].

SMUG1 cannot compensate fully for UNG2 in class-switch recombination (CSR) and somatic hypermutation (SHM) [74–76]. Although a genetic study by Dingler *et al*. showed that SMUG1 was able to access AID-induced U:G lesions in immunoglobulin genes and could partially substitute for UNG2 in both CSR and SHM [77], no contribution of SMUG1 was seen when UNG2-initiated BER and the mismatch repair pathway were functional [77]. Thus, although there may be differences in SMUG1 activity in different organisms, the lack of truly comparable studies, e.g. comparing the same cell types and endpoints, makes it difficult to firmly conclude whether there are qualitative differences between species or if the apparent discrepancies reflect different conditions.

## SMUG1 in cancer

Multiple single-nucleotide polymorphisms (SNPs) in SMUG1 have been suggested as independent prognostic factors associated with poor survival in patients with cancer of the colon [78, 79], breast [80], bladder [81], and cervix [82], but the mechanisms underlying these associations are not known. From studies in cancer cell lines, however, conditions where SMUG1 may influence cellular responses to chemotherapy are emerging.

**Figure 2.**
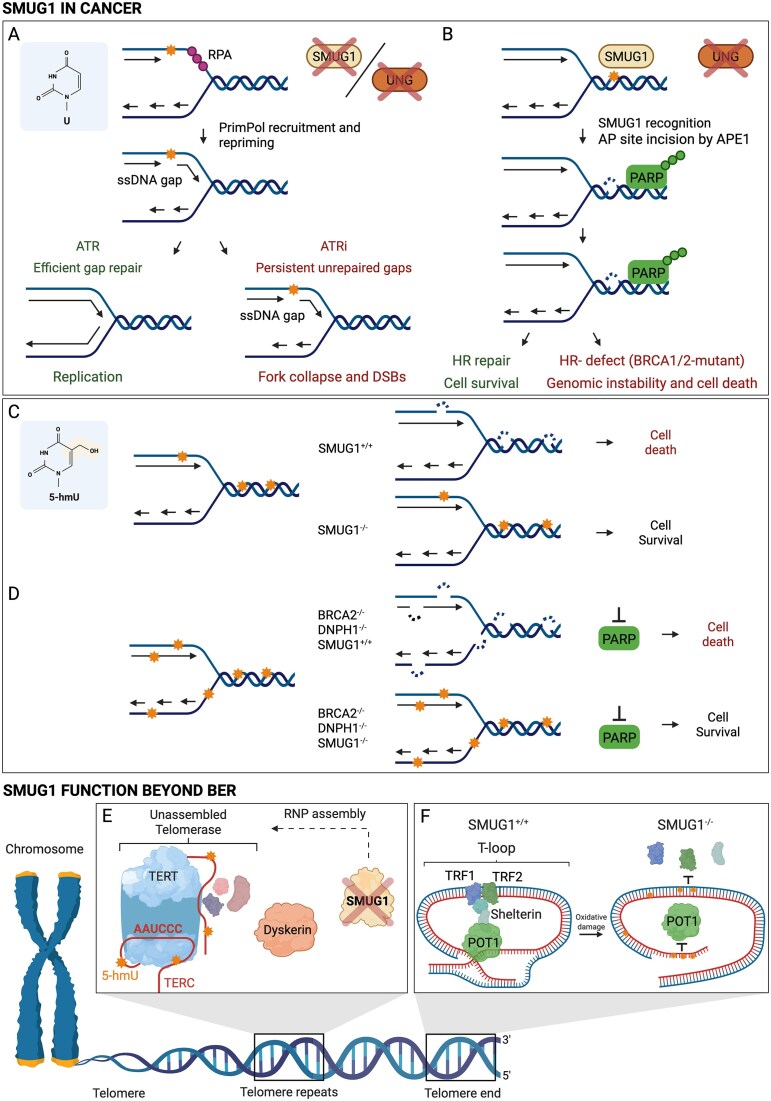
Multifaceted roles of SMUG1 in genome stability, replication stress, and RNA regulation. A–B: Uracil-driven replication stress and synthetic lethality. (**A**) UNG deficiency induces uracil accumulation and replication stress in U2OS cells. Loss of UNG2 leads to excessive incorporation of uracil during replication, resulting in replication fork slowing, PrimPol-mediated ssDNA gap formation, and increased sensitivity to ATR inhibitors (ATRi). In U2OS cells, SMUG1 depletion contributed to cellular uracil burden and ATRi response. (**B**) SMUG1-dependent toxicity in homologous recombination (HR)-deficient cells. In *TP53^−/^*^−^ hTERT RPE-1 cells, SMUG1 promotes uracil excision in the absence of UNG, leading to replication stress characterized by single-strand breaks and PARP1 activation. These lesions require HR for repair. In BRCA1/2-deficient cells, which lack effective HR, SMUG1 activity renders uracil incorporation toxic, suggesting therapeutic potential for targeting SMUG1 in HR-deficient tumours. (C, D) SMUG1-mediated processing of 5-hydroxymethyluracil (5-hmdU) and synthetic lethality. (**C**) SMUG1 promotes 5-hmdU toxicity through BER intermediates. Knockdown of SMUG1 in SW480 and HeLa cells reduces sensitivity to 5-hydroxymethyl-2′-deoxyuridine (5-hmdU), indicating that SMUG1 excises this modified base and initiates a BER process that generates cytotoxic intermediates. Further blocking downstream BER steps enhances toxicity, underscoring SMUG1’s role in converting base analogues into DNA damage. (**D**) SMUG1-dependent synthetic lethality in *DNPH1^−/−^BRCA2^−/−^* cells. DNPH1 loss leads to accumulation of 5-hmdU monophosphate, which is incorporated into DNA. In BRCA2-deficient cells reliant on PARP1 for repair, SMUG1 excision of 5-hmdU generates toxic abasic sites. PARP1 inhibition exacerbates this effect. Interestingly, SMUG1 depletion rescues sensitivity, indicating SMUG1 is essential for 5-hmdU–driven cytotoxicity in HR-deficient contexts. (E, F) SMUG1 function beyond DNA repair. (**E**) SMUG1 interacts with RNA-modifying enzyme DKC1 and influences telomere stability. SMUG1 binds to Dyskerin (DKC1), which catalyzes pseudouridylation of various RNAs including rRNA and the telomerase RNA component (hTERC). In SMUG1-deficient cells, telomeric chromatin immunoprecipitation (ChIP) shows reduced association of Shelterin complex proteins, particularly POT1, likely due to DNA lesions impairing proper telomere protection. (**F**) SMUG1 loss disrupts telomere end protection. *SMUG1^−/−^* cells show decreased recruitment of Shelterin proteins, especially POT1. This is likely due to DNA lesion accumulation at telomeres, disrupting proper telomere capping.

Recently, we have seen a renewed momentum surrounding SMUG1, fuelled by its emerging role as a key modulator of sensitivity to nucleoside analogues under conditions of replication stress (RS) in cells deficient in homologous recombination (HR). While misincorporated uracil is not considered cytotoxic or mutagenic, Saxena *et al*. used DNA fibre assays in U2OS cells to show that uracil misincorporation slows replication forks and triggers the formation of PRIMPOL-dependent ssDNA gaps, thereby increasing ATR inhibitor (ATRi) sensitivity (Fig. 2A) [83]. PRIMPOL is a primase that can reinitiate DNA synthesis downstream of stalled replication forks, thereby promoting replication restart but leaving behind ssDNA gaps. Interestingly, they found that misincorporated uracil itself induced RS and that UNG2-depletion exacerbated RS and ATRi sensitivity by increasing the load of genomic uracil. Interestingly, depletion of SMUG1 similarly exacerbated ATRi sensitivity in this study. This was an unexpected finding, as SMUG1 is not described to be cell cycle regulated or associated with the replisome. Yet, genetic interactions between SMUG1 and PRIMPOL have been observed by several groups [84–86], with some suggesting that SMUG1 contributes to ssDNA gap formation [86].

Similarly, slowing down of replication fork progression with subsequent accumulation of post-replicative ssDNA gaps accompanied by PARP inhibition led to sensitivity after siRNA-mediated depletion of UNG in *TP53^−/^*^−^ hTERT RPE-1 cells. Consistent with UNG being the replisome-associated uracil-DNA glycosylase, depletion of SMUG1 alone was not associated with ssDNA gap formation. However, co-depletion of SMUG1 rescued both the ssDNA gap formation and PARP inhibitor sensitivity in UNG-depleted cells. Together these studies suggest that SMUG1 may also operate close to the replication fork and that, in the absence of UNG, processing of uracil or uracil derivatives by SMUG1 generates post-replicative DNA repair intermediates requiring HR for repair (Fig. 2B) [87].

SMUG1 has been shown to influence cancer cell survival [88], especially in response to nucleoside analogues. Pettersen *et al*. provided initial mechanistic insights: in HR-proficient SW480 cells, and to a lesser extent HeLa cells, knockdown of SMUG1 reduced sensitivity to the nucleoside 5-hydroxymethyl-2′-deoxyuridine (5-hmdUrd), suggesting that SMUG1 generates toxic BER intermediates by processing these nucleoside analogues after their incorporation into DNA (Fig. 2C) [35, 43]. Furthermore, inhibition of repair downstream of SMUG1 exacerbated the toxicity of these intermediates. In line with this, Nagaria and co-workers showed that SMUG1, but not UNG2, was required for RF restart after replication stress induced by 5-FU [89].

In a study by Fugger *et al*., however, knockout of SMUG1 suppressed sensitivity to PARP1 inhibition in *DNPH1^−/−^* and *BRCA2^−/−^* cells (Fig. 2D) [90]. DNPH1 is a nucleotide pool sanitizing enzyme that hydrolyzes 5-hmdUrd monophosphate, preventing its accumulation. When DNPH1 is absent, 5-hmdU accumulates and is incorporated into DNA. In HR-deficient cells, such as those lacking functional BRCA2 and therefore heavily reliant on PARP1 for DNA repair, PARP1 inhibition leads to DNA break formation and cell death. The observed suppression of sensitivity upon genetic SMUG1 perturbation suggests that SMUG1-mediated excision of 5-hmdU created toxic DNA lesions in this setting.

Under a hypothesis that enzyme inhibition recapitulates enzyme knockdown and activation overexpression, one may expect a possible utility for SMUG1 activators or inhibitors in adjuvant settings, e.g. to sensitize HRD tumour to PARPi or ATRi, respectively [85, 90–92]. However, a pioneering study on pharmacological SMUG1 modulation in human cancer cell lines suggests that this might be simplistic. The SMUG1 activator SU0547 rescued cells from 5-hmdUrd-induced toxicity at ∼3 µM concentration [91], thus recapitulating the effects of genetic SMUG1 downregulation and knockout, rather than overexpression [43, 51, 90, 93]. In the absence of 5-hmdUrd, the activator conferred toxicity to cell lines such as MCF7 and SUM149PT at concentrations of the same order of magnitude that activated SMUG1 *in vitro* [91], suggesting that processing of endogenously occurring SMUG1 substrates may induce cytotoxicity. This demonstrates that there is much still to be elucidated about how SMUG1 functions to maintain genomic stability in the face of cancer therapy and endogenous replication stress. Indeed, several chemotherapeutics in current use induce SMUG1 substrates (Table 1). Thymidine synthase inhibitors like methotrexate and raltitrexed, as well as dUTPase inhibitors that are in clinical trial phases, lead to uracil incorporation. Standard chemotherapy regimens like FOLFOX, FOLFIRINOX, and FOLFIRI lead to 5-FU incorporation. Trifluridine and the 5-FU prodrug NUC-3373 are metabolized into uracil-derived nucleotides that can be incorporated into DNA [94]. While 5-FU is known to be excised by SMUG1, trifluridine is expected to be a poorer substrate, given its bulkier trifluoromethyl substitution at the 5-position compared with smaller uracil derivatives such as 5-hmU. It may therefore be possible that it might stall repair at the recognition level or through other molecular targets. In addition, SMUG1 may interact with several other nucleoside or nucleotide analogues. For example, cytarabine, azacitidine, and its deoxyribose analogue decitabine are approved nucleoside analogues that contain structural modifications within both the sugar and the cytosine base. However, since SMUG1 specifically recognizes uracil generated by the deamination or hydrolysis of cytosine, it is unlikely that the enzyme efficiently recognizes or removes these analogues from DNA.

## SMUG1 connecting BER and RNA metabolism

The correlation between SMUG1 and cell survival may also reflect an indirect role of SMUG1 in cancer development. Jobert *et al*. showed that SMUG1 interacts with DKC1, an enzyme responsible for catalysing the pseudouridylation of specific uridine residues in RNA (Fig. 2E) [22]. DKC1 plays a critical role in the biogenesis of ribosomes, spliceosomal small nuclear ribonucleoproteins, microRNAs, and the telomerase ribonucleoprotein complex. Thus, the interaction with DKC1 brings SMUG1 in contact with RNA species processed by DKC1 such as rRNA [22, 23] and the telomeric RNA component hTERC [25].

Interestingly, independent confirmation of SMUG1’s role in rRNA processing [22] was recently provided where depletion of SMUG1 in mouse embryos was accompanied by unstable 47S pre-rRNA, which in turn reduced mature 18S and 28S rRNA, and a failure to develop at a normal pace [27]. How this finding is compatible with SMUG1 knockout mice having no obvious developmental defect remains to be determined [21, 67]. It might suggest that maternal stores of ribosomal RNA and other enzymes compensate during gestation. These experiments may point to potential vulnerabilities, but the underlying principles by which SMUG1 impacts RNA maturation or RNA quality control remain to be clarified. We previously showed that, in human cells, SMUG1 is part of a gene regulatory network involving a regulatory loop with the *let-7b-5p* miRNA that influences survival and treatment response in several cancers [29]. SMUG1 physically interacts with *let-7b-5p* and other members of the *let-7* microRNA family *in vivo* and regulates *let-7b-5p* expression levels. Given the well-established tumour-suppressive role of *let-7* microRNAs [95], the association between SMUG1 expression and breast cancer prognosis may, at least in part, be independent of its canonical role in DNA repair.

Similarly, while SMUG1 is crucially important for maturation and stability of the telomeric RNA component hTERC in HAP1 cells [25] and primary bone marrow cells in mice [26], other cell types do not appear to critically depend on SMUG1 in this regard. It should be kept in mind that the SMUG1 DNA repair function might affect telomere homeostasis [24, 26]. In *SMUG1^−/−^* cell lines, telomere chromatin immunoprecipitation (ChIP) assays revealed a reduced association of Shelterin-complex proteins, with the most notable decrease observed for POT1, which plays a key role in capping telomere ends. A likely explanation is that the absence of SMUG1 leads to the accumulation of DNA lesions at telomeres, thereby hindering POT1 binding (Fig. 2F) [25]. Consistently, complementation experiments performed by Kroustallaki *et al*. suggested that neither SMUG1 mutant defective in the RNA processing or BER functions were able to fully reconstitute the telomere maintenance defects. Thus, the DNA repair function of SMUG1 also contributes to telomere maintenance in HAP1 cells.

Another connection where SMUG1 might link RNA and DNA metabolism is through the proposed role in epigenetic control of gene expression (reviewed by Lirussi *et al. *[29]). In mouse embryonic stem cells, TET enzymes were found to induce 5-hmU to similar levels as 5-caC but appeared to do so mainly by direct oxidation of thymine [4]. Recently, loss of SMUG1 was shown to modulate gene expression programs in the mouse, where 5-hmU accumulation was associated with reduced expression of genes regulated by certain A/T-rich promoter genes [28]. A direct role of SMUG1 in regulating these promoters was suggested by ChIP at the promoters of differentially expressed genes. Whether a similar mechanism is relevant in cancer cells remains to be defined, but a role for SMUG1 in gene regulation might explain other associations with SMUG1 and metabolic homeostasis [26, 96]. Interestingly, somatic gain-of-function mutations in TET2, which seems to preferentially oxidize thymine, were recently identified in AML patients, where SMUG1 knockout yielded a higher hmU/hmC ratio [97].

## Conclusions and future outlook

The current evidence points to potential therapeutic opportunities arising from exploiting SMUG1-mediated BER to induce synthetic lethality in combination with PARP and ATR inhibitors, particularly in BRCA1/2-deficient tumours. However, the first pharmacological targeting of SMUG1 in cancer cell lines showed that SMUG1 activation reduces toxicity to the same degree as SMUG1 knockouts. This suggests that key mechanistic questions remain unresolved and addressing these will be critical for defining whether, and in which contexts, SMUG1 modulators might have clinical utility.

One of the most urgent questions is which SMUG1 substrate, or the products of SMUG1-mediated BER, generates lesions that require HR or ATR for repair. Several of the recent studies employed assays reliant on one or more nucleoside analogues (EdU, CldU, BrdU, IdU) to monitor replication. EdU has been shown to be a substrate for nucleotide excision repair [66]. There is data showing that the halogenated substituents may be substrates for SMUG1; A bromo substituent and hydroxymethyl are larger than any of the other halogen substituents, suggesting that SMUG1 may be able to excise all halogenated thymidine analogues used to interrogate replication fork dynamics. Moreover, an electron pulling effect is observed for F > Cl > Br > I. This is expected to introduce a partial positive charge on the adjacent C atom and from there to every second carbon. This suggests that F-substituted bases should be easier to excise than bases with less electron pulling adducts. Several groups have demonstrated that FdU is a substrate for both UNG and SMUG1 [20, 42, 43]. Similarly, BrdU- and IdU-containing oligonucleotides are shown to be SMUG1 substrates *in vitro* [44]. CldU has, to the best of our knowledge, not been tested as a substrate for SMUG1, but CldU was recently shown to be an UNG substrate, and processing of CldU by UNG in the template strand was shown to trigger replication fork collapse in an X-ray cross-complementing protein 1 (XRCC1)-deficient background [98]. Thus, it is difficult to exclude the possibility of assay interference when these reagents are used to monitor replication forks following exposure to the nucleoside analogues (FdU, dU, 5-hmdU). Adding to the complexity of interpretation, the exact identity of the lesion(s) providing toxicity is not ascertained with direct measurements, using instead indirect immunostainings or Comet assays coupled to DNA glycosylases with broad substrate specificities. As toxic lesions might not necessarily require incision, it would be important to acquire biophysical measurements or single-molecule analyses where all UNGs and substrates were tested against one another under a range of conditions.

Another important question relates to how SMUG1 distinguishes between thymine and uracil, facilitated by a well-ordered water molecule in the active site was observed in the crystal structures of both xSMUG1 and GmeSMUG1. While this mechanism is assumed to be conserved in hSMUG1, no crystal structure currently exists to confirm it. Looking ahead, resolving the structure of hSMUG1 remains an important goal for understanding its substrate specificity and function. Biochemical studies and single-molecule studies to clarify whether SMUG1 binding at AP sites is strong enough to cause replication block.

With a possible cancer relevance of SMUG1 emerging, it will be important to develop technologies that enable precise tracking of SMUG1 localization *in vivo*, particularly in relation to the replication fork. Notably, SMUG1 was not detected at replication forks using isolation of proteins on nascent DNA [87]. It is possible that SMUG1 acts only transiently or functions outside of the core replication machinery. Interestingly, the same study reported that the interplay between UNG2 and SMUG1 is regulated by nuclear nicotinamide adenine dinucleotide (NAD^+^), which maintains UNG2 at RFs and restrains SMUG1’s chromatin binding, thereby preventing the generation of SMUG1-mediated cytotoxic lesions [87].

In conclusion, SMUG1 is a versatile and multifaceted protein that bridges DNA repair, RNA metabolism, and gene regulation. As such, understanding its diverse roles will not only refine our view of genome maintenance mechanisms but may also unveil novel opportunities for therapeutic intervention in cancer.

## Funding

This research was funded by the Norwegian Cancer Society, grant number 223314 (H.L.N) and grant no. 272410 (N.P.M). This work was partially supported by the Research Council of Norway through its Centres of Excellence scheme, Project Number 332713. T.V. acknowledges funding from the Norwegian Research Council (grant Nos. 303369 and 353112). This project has received funding from the Innovative Medicines Initiative 2 Joint Undertaking (JU) under grant agreement No 875510 (N.R., M.M.). The JU receives support from the European Union’s Horizon 2020 research and innovation programme, EFPIA, Ontario Institute for Cancer Research, Royal Institution for the Advancement of Learning McGill University, Kungliga Tekniska Högskolan, and Diamond Light Source Limited.

## Data Availability

No new datasets were generated or analysed. Source data for figures are available upon reasonable request.
